# Predictors of regular physical activity behavior and quality of life in post-menopausal Iranian women based on the multi-theory model

**DOI:** 10.25122/jml-2021-0073

**Published:** 2022-03

**Authors:** Nooshin Yoshany, Mohammad Ali Morowatisharifabad, Manoj Sharma, Sara Jambarsang, Narjes Bahri, Reza Sadeghi, Fahad Hanna

**Affiliations:** 1. Department of Health Education and Promotion, Social Determinants of Health Research Center, School of Public Health, Shahid Sadoughi University of Medical Sciences, Yazd, Iran; 2. Aging Health Department, School of Public Health, Shahid Sadoughi University of Medical Sciences, Yazd, Iran; 3. Environmental & Occupational Health, School of Public Health, University of Nevada, Las Vegas, United States; 4. Department of Biostatistics and Epidemiology, School of Public Health, Shahid Sadoughi University of Medical Sciences, Yazd, Iran; 5. Department of Midwifery, Faculty of Medicine, Social Determinants of Health Research Center, Gonabad University of Medical Sciences, Gonabad, Iran; 6. Department of Public Health, School of Public Health, Sirjan School of Medical Sciences, Sirjan, Iran; 7. Public Health Program, Faculty of Health Sciences, Torrens University Australia, Melbourne, Australia

**Keywords:** adaptation to menopause, multi-theory model, physical activity, menopause, quality of life

## Abstract

This research aims to identify the predictive factors related to the initiation and sustaining of regular physical activity behaviors and their influence in adapting to menopausal symptoms. The study uses the multi-theory model (MTM) as the conceptual framework. The descriptive cross-sectional research was conducted on 200 post-menopausal women aged 45-55 years. All participants were referred to health centers, where they completed a three-part questionnaire involving: demographic information, a questionnaire on the influence of regular physical activity on the onset and sustaining of menopause using the MTM, and a standard questionnaire of menopausal quality of life. Data were collected, managed, and analyzed using SPSS 20 and AMOS 23 software. This study reveals that behavioral confidence and changes in the physical environment with coefficients of 0.55 and 0.14 respectively had a direct and significant impact on the initiation of regular physical activity-related behaviors during menopause. The construct of participatory dialogue had no significant impact on the initiation of regular physical activity-related behaviors and menopause. Regarding the sustainability of regular physical activity, the emotional transformation construct with a coefficient of 0.73 and change in social environment construct with a coefficient of 0.11 directly and significantly impact keeping regular physical activity behaviors due to menopause. Therefore, interventions based on the MTM can prove very useful for this specific population. In addition, the use of constructs validated by this study will especially be useful in producing evidence-based intervention for the target population.

## INTRODUCTION

There are different methods of decreasing menopausal symptoms, such as hormone therapy, decreasing caffeine and alcohol consumption, changing lifestyles, and physical activity [1]. Since hormone therapy does not apply to all women because it is associated with different complications, non-pharmacological approaches, one of which is participation in sports activities, have received more attention [2]. Including regular physical activity in the daily life plan is a significant factor in sustaining and promoting health [3]. Women who participate in regular physical activity have a higher bone density [4, 5] and improved sleep quality [6]. Also, exercise plays an important role in stabilizing normal blood circulation and other physiological activities during menopause [3]. Some of the issues influencing the quality of life among postmenopausal women are pelvic floor muscle function disorders, urinary problems, and sexual dysfunction [7]. Kegel exercises are recommended to strengthen pelvic floor muscles, which improve urinary incontinence in women by increasing the blood supply in the urinary and genital areas [8]. Kegel exercises are free and uncomplicated, and can be performed at any time of the day [9]. Regular swimming exercise effectively improves body composition, physical strength, and blood lipids in middle-aged women [10]. Exercise and regular physical activity release the endorphin hormone and thus reduce discomfort and pain, increasing pleasant emotions.

Regular physical activity positively impacts happiness in post-menopausal women [11]. Several studies reported the positive impacts of regular physical activity on vasomotor symptoms [2, 12–14] and on decreasing amnesia, aggression, and anxiety [15].

One of the most appropriate strategies to control menopausal complications and improve quality of life is designing interventions and educational programs. This could be accomplished by utilizing models and theories of behavior to help in predicting and changing individual, interpersonal, and community behaviors. The use of factors from models and theories can assist in predicting the frequency of occurrence and change of behaviors in various cultures. It can also lead to efficient planning of programs [16]. One of the most applicable and efficient models in the literature is the multi-theory model (MTM) of behavior change. It is a cost-effective model with flexible structures. It applies to many behaviors, including recurring and long-term behaviors and behaviors that are performed once. This model was designed by Manoj Sharma, and it is culturally sensitive and thus applicable to various cultures.

The MTM has two significant parts: (1) initiation of behavior change and (2) sustaining that behavior. According to MTM, the initiation stage utilizes participatory dialogue, behavioral confidence, and changes in the physical environment, while the sustaining stage utilizes emotional transformation, practice for change, and change in the social environment [17]. This model has been successfully applied in the prediction of behaviors such as regular physical activity [18], fruit and vegetable consumption [19], water consumption instead of gaseous and sweet drinks [20], and consumption of low-volume meals [21]. Regular physical activity plays a significant role in physical health [22]; however, recent studies showed a marked shift in lifestyle toward inactivity and a reduction in daily energy consumption [23, 24].

Various factors impact regular physical activity in the design of educational interventions to improve life quality during menopause. It is important to identify the factors associated with initiating and sustaining regular physical activity behaviors essential to controlling menopausal complications. Therefore, this study aims to identify the predictors of initiating and sustaining regular physical activity behaviors. These will involve exercise, strengthening the pelvic floor muscles, walking, and swimming, as indicated in the MTM.

## MATERIALS AND METHOD

This descriptive cross-sectional research was conducted in Yazd, central Iran, in 2019. The protocol was developed after receiving the clinical trial code (IRCT registration number: IRCT20190206042640N1) used in a study by Morowatisharifabad *et al.* [25]. Subjects comprised 200 post-menopausal women who were referred to health centers in Yazd. Study participants were required to fulfill six criteria as follows:

• Post-menopausal women with a minimum of one year and a maximum of five years of menopause;
• Normal menopause;
• Lack of physical illness and sobriety disorder;
• Age range of 45–55 years;
• Not having addiction;
• Willingness to participate in the research by completing the informed consent form.

A list of health centers in Yazd city was provided, compiled, and categorized by the socioeconomic status of clients: low, middle, and high socioeconomic status. Then two centers were randomly chosen from each group, areas with low, middle, and high socioeconomic status. First, the list of women aged 45–55 years covered by the centers was extracted. Then, according to the number of clients of each center (Center1: 30, Center2: 35, Center3: 38, Center4: 32, Center5: 34, Center6: 31), they were chosen by simple random sampling from each center. Finally, the participants completed three questionnaires involving demographic information, a researcher-developed questionnaire of regular physical activity behaviors influencing menopause compliance using the MTM, and a standard questionnaire of menopause quality of life (MENQoL).

Before completing the questionnaire, participants were informed about the research objectives, and other information related to confidentiality and anonymity was explained. The researchers developed a questionnaire with several subscales using the MTM. These were validated by 14 professors and experts in health education and reproductive health for face and content validity. The reliability and validity of the questionnaire were computed using the responses of 21 post-menopausal women. The intra-cluster correlation coefficient that resulted from this computation was 0.92. The demographic questionnaire comprised 26 questions, which included age, age of last menstruation, age of first menstruation, age of first pregnancy, age of last pregnancy, marriage age, age of menopause of mother or sister, regular or irregular menstrual cycles, education, occupation, income, weight, height etc. The questionnaire developed by the researchers involved 34 questions which assessed several constructs based on the multi-theory model (MTM). For example, the construct of participatory dialogue covers how efficient walking is in decreasing hot flashes.

It also covers the extent to which the opposition of family members and those around the participants influence the use of the swimming pool. The construct of behavioral confidence included three questions. For example, how sure the participants were that despite not being enjoyable, they still do the exercise of strengthening the pelvic floor muscles. The construct of change in the physical environment included 4 questions, *e.g.*, how sure the participants are to be able to find a suitable space for walking. The emotional transformation construct included nine questions, *e.g.*, how sure the participants are to encourage themselves to go to the pool. The construct of practice for change included six questions, such as how sure the participants are that they can record their pelvic floor exercises on a weekly notebook, to monitor their pelvic floor muscle strengthening. The construct of change in the social environment comprised five questions, for instance, how sure the participants are to get help from their family members in going to the pool. The answer to these items was scored as 0, 1, and 2, with "I'm not sure at all" being 0, "partly sure" 1, and "I'm pretty sure" 2. Participants were to write down the situation weekly in a notebook, with a score of 0 points for "I'm not sure", a score of 1 for "somewhat sure", and a score of 2 for "I'm pretty sure". The initiation construct consisted of 3 questions, for instance, how likely it is to start walking 30 minutes a day next week. Finally, the behavior sustaining construct included three questions, for example, how likely it is to put 30 minutes of walking into their daily schedule from now on, where "not likely" was scored 0, "somewhat likely" was scored 1, and "quite likely" was 2.

The menopausal quality of life questionnaire was designed and standardized by Hilditch *et al.* at the University of Toronto, Canada. The questionnaire consisted of 29 questions with four conditions of vasomotor (3 questions), psychosocial (7 questions), physical (16 questions), and sexual (3 questions). Answers were measured on a 6-point Likert scale ranging from 1 (I don't have symptoms) to 6 (very severe). The total score of the questionnaire was between 29 and 174, with a lower score indicating a better quality of life [26, 27]. Fallahzadeh *et al.* reported this questionnaire's reliability to have Cronbach's alpha coefficient of 0.85 [28].

After entering the data information in SPSS software version 18, the relationships among variables were assessed in a path analysis model using the AMOS software version 23. Descriptive indices of mean and standard deviation were utilized to summarize the dimensions score of the questionnaire, and path analysis to estimate factor loads for participatory dialogue, behavioral confidence, changes in the physical environment, emotional transformation, practice for change and change in a social environment on quality of life. Goodness-of fit (GFI), comparative fitting (CFI), and mean approximation error square (RMSEA) indices were applied to measure the adequacy of the model.

## RESULTS

The mean (standard deviation) age, menopause age, and menopausal age of first-degree relatives were 52.61 (2.03), 49.58 (2.04), 49.44 (2.54), respectively. About 91.5% (183) had a history of regular menstrual cycles. A total of 76% of women (152) and 70.5% of their spouses (141) had diplomas and sub-diplomas. A total of 80% of women were housewives (169), and most of their spouses were retired 46.5% (93). The mean (standard deviation) BMI was 29.64 (4.29). The majority of women with middle socioeconomic status had regular periods (94 (47%)). The majority of women with associate and bachelor's degrees were in high socio-economic status (19 (9.5%)). 88 (44%) women with middle level of socio-economic status were housewives (Table 1).

**Table 1.** Frequency and absolute distribution of research units based on some demographic variables.

	Variable	Socioeconomic status			
	Classification →	Low	Middle	High	Total
Variable	Classification ↓	Frequency (Percent)	Frequency (Percent)	Frequency (Percent)	Frequency (Percent)
**Types of menstrual cycles**	Regular	56 (28)	94 (47)	33 (16.5)	183 (91.5)
	Irregular	5 (2.5)	10 (5)	2 (1)	17 (8.5)
**Women's educational levels**	Illiterate	12 (6)	4 (2)	0 (0)	16 (8)
	Diploma and lower	46 (23)	90 (45)	16 (8)	152 (76)
	Associate and bachelor's degree	3 (1.5)	10 (5)	19 (9.5)	32 (16)
**Occupation**	Employed	4 (2)	6 (3)	6 (3)	16 (8)
	Housewife	53 (26.5)	88 (44)	19 (9.5)	160 (80)
	Retired	4 (2)	10 (5)	10 (5)	24 (12)
**Husbands' educational levels**	Illiterate	10 (5)	6 (3)	0 (0)	16 (8)
	Diploma and lower	51 (25.5)	75 (37.5)	15 (7.5)	141 (70.5)
	Associate and bachelor's degree	0 (0)	17 (8.5)	14 (7)	31 (15.5)
	Master's and PhD. degrees	0 (0)	6 (3)	6 (3)	12 (6)
**Husband's occupation**	Unemployed	0 (0)	2 (1)	2 (1)	4 (2)
	Self-employed	38 (19)	42 (21)	10 (5)	90 (45)
	Government job	0 (0)	10 (5)	3 (1.5)	13 (6.5)
	Retired	23 (11.5)	53 (25)	20 (10)	93 (46.5)

The mean, standard deviation, as well as minimum and maximum attainable scores from various constructs of the MTM are reported in Table 2.

**Table 2.** Mean score of MTM constructs in physical activity behaviors.

Variable	Mean (SD)	Maximum obtained score	Minimum obtained score
**Participatory dialogue**	7.24 (9.30)	29	-26
**Behavioral confidence**	2.72 (1.14)	6	0
**Changes in the physical environment**	5.35 (2.09)	8	0
**Emotional transformation**	10.43 (3.46)	18	1
**Practice for change**	3.81 (2.31)	9	0
**Changes in the social environment**	4.20 (2.07)	10	0

**Figure 1.**
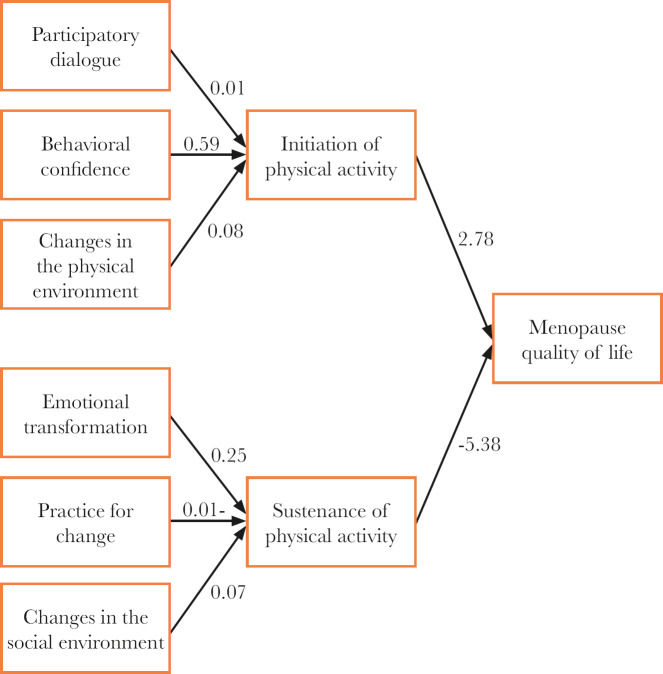
Output path analysis of the effective factors on initiation and sustenance of the physical activity and quality of life (numbers represent standardized coefficients of the model).

Table 3 and Figure 1 (numbers in the figure represent standardized estimates of path analysis model) indicate that there is a significant relationship between behavioral confidence and changes in the physical environment with the initiation of behavior (p <0.001 and p=0.013, respectively). There was a significant relationship between emotional transformation and changes in the social environment with the sustainability of regular physical activity (p<0.001). There was a significant relationship between the initiation and sustainability of behavior with quality of life (p<0.001 and p=0.017, respectively). Furthermore, for one unit increase in the initiation score of regular physical activity behavior, the quality of life score increased by 2.77. For one unit increase in the sustaining score of regular physical activity behavior, the quality of life score decreased to 5.37. Goodness- of-fit index (GFI) was 0.88, the comparative fit index (CFI) was 0.86, and the root mean square error of approximation (RMSEA) was 0.21.

**Table 3.** MTM constructs coefficients in the initiation and sustaining of physical activity behaviors in accordance with menopause.

Variables			Estimate	S.E.	C.R.	P-value	Standardized coefficient
**Initiation of physical activity behavior**	←	Participatory dialogue of physical activity behavior	.00	.00	.68	.494	.04
**Initiation of physical activity behavior**	←	Behavioral confidence of physical activity behavior	.59	.06	9.63	<0.000	.55
**Initiation of physical activity behavior**	←	Changes in the physical environment of physical activity behavior	.08	.03	2.47	.013	.14
**Sustenance of physical activity behavior**	←	Emotional transformation of physical activity behavior	.25	.01	15.30	<0.000	.73
**Sustenance of physical activity behavior**	←	Practice for change of physical activity behavior	-.00	.03	-.24	.81	-.01
**Sustenance of physical activity behavior**	←	Changes in the social environment	.06	.02	2.38	.017	.11
**Menopause quality of life**	←	Initiation of physical activity behavior	2.77	1.32	2.09	.036	.14
**Menopause quality of life**	←	Sustenance of physical activity behavior	-5.37	1.35	-3.97	<0.000	-.26

S.E. – Standard error; C.R. – critical ratio.

## DISCUSSION

Behavioral confidence and changes in the physical environment with coefficients of 0.55 and 0.14 had a direct and significant impact on the initiation of regular physical activity-related behaviors in adapting to menopause. The construct of participatory dialogue had no significant impact on the initiation of regular physical activity-related behaviors.

A participatory dialogue that refers to a person's attitude toward the benefits and barriers of doing a behavior had no significant effect on the initiation of regular physical activity in post-menopausal women. This means that knowing the benefits of exercise and the presence of barriers to exercise did not affect the initiation of regular physical activity in women. This finding is in line with the study of Nahar *et al.* [29] regarding the application of an MTM in initiating and sustaining regular physical activity in medical students with osteoporosis. Furthermore, in his study on predicting osteoporosis-preventive behaviors based on the health belief model, Vahedian [30] reported that perceived benefit and barriers constructs are not significant predictors of regular physical activity behavior in women under 60 years of age. Nevertheless, Nahar *et al.*, [31] and Hayes *et al.* [18] in a study on the use of MTM in prediction of initiation and sustainability of regular physical activity in African American women over 18 years old, reported inconsistent results with the present study. This could be attributed to the difference in research design, population, culture, and race. In this study, the insignificant effect of participatory dialogue construct in initiating regular physical activity behavior could be due to high awareness of women regarding the benefits of exercise and its effect on menopausal symptoms, usually these symptoms are so annoying that they lead people to look for different solutions. Today, one of the sources people refer to answer their questions is cyberspace and the internet, so people know the benefits of appropriate behavior. And it seems that things like family opposition, cost, and time, cannot be a big obstacle to women's physical activity. And if people really want to exercise, they can succeed with the least cost and proper planning. Besides, within the population of the current study, which is post-menopausal women, just as within students with osteoporosis [29], because of their situation, regular physical activity is usually recommended by the health team, so their awareness of the benefits of behavior is high, and as a result, this construct is not a strong predictor of the initiation of behavior.

In the current study, the behavioral confidence construct was a strong and significant predictor of the initiation of regular physical activity behavior, meaning that the more confident a person was in his or her ability to perform regular physical activity behavior, the more likely he or she was to initiate the behavior, which was consistent with other studies [29, 31]. Also, Sadeghi Tajdanoo *et al.* [32] reported in their research that self-efficacy is an important and significant determinant in doing regular physical activity behavior. However, Asare *et al.* [33], in their study on the application of an MTM in human papillomavirus vaccination in Ghanaian adolescents, acknowledged that only the perceived belief and change in physical environment predict the initiation of vaccination, which is not consistent with the present study. The reason for this inconsistency could be a difference in the nature of the behaviors studied, and it seems that behavior such as regular physical activity because it is long-term in nature, is more influenced by behavioral confidence than vaccination. Nevertheless, for vaccination, a person's belief in the benefits and barriers of vaccination and the availability of environmental access are the most important predictors [33]. In the present study, the construct of change in the physical environment was a significant predictor of the initiation of regular physical activity behavior. This means that the more a woman had access to sports facilities and places in her living environment, the more likely she was to initiate regular physical activity. Nahar *et al.* also acknowledged that change in the physical environment is a significant predictor of the initiation of regular physical activity among students [31].

Also, Motl *et al.* confirmed the importance of the physical environment, available facilities, and equipment in increasing the physical activity of adolescent girls [34]. Pirasteh *et al.* reported that improving the safety of the living environment has a positive effect on the promotion and continuation of exercise and physical activity in adolescents [35]. Peyman *et al.* also reported the environment as the strongest predictor of physical activity in their study [36]. It seems that the presence of environmental facilities and accessibility, security and beauty, such as the existence of a safe place for women to walk without traffic and street disturbances, play an important role in encouraging and motivating them to do regular physical activity. According to the path analysis test, regarding behavior sustainability, the construct of emotional transformation with a coefficient of 0.73 and the construct of change in the social environment with a coefficient of 0.11 had a direct and significant effect on the sustainability of regular physical activity. However, the construct of practice for change did not have a direct and significant effect on the sustainability of regular physical activity.

In this study, emotional transformation, which means motivating oneself and overcoming doubts in performing regular physical activity, was a significant predictor in sustaining and continuing women's physical activity. This means that the more people were able to direct their emotions and feelings to exercise and overcome it without doubt, the more they could continue exercising. In the study of Nahar *et al.* [31], a significant predictive emotional change in the continuation of students' physical activity was also reported. Hayes *et al.* [18] also acknowledged that emotional transformation predicts continued physical activity in African American women. The construct of emotional transformation is derived from the theory of emotional intelligence, and the concept of emotional intelligence refers to how people adapt and succeed in life situations.

Emotional intelligence theory justifies a wide range of abilities to recognize and use emotions [37]. In general, emotional intelligence is an important factor in determining life success and mental health. This is because it affects the ability of individuals to effectively cope with environmental pressures and barriers [38]. In explaining this finding, it can be said that having high emotional intelligence can positively adapt a person to the needs, pressures, and obstacles in life. People who have high emotional intelligence and are able to properly assess and regulate their emotions can better overcome their doubts and focus their emotions on continuous regular physical activity. Practice for change means monitoring daily behavior and focusing on the goal in the confronted obstacle and the ability to change the program if confronted with a problem. The practice for change construct did not predict the sustainability of behavior meaning that steps or actions such as putting a pool bag in the car to facilitate and remind you to go to the pool or to record daily sports activities in a notebook, did not predict the continuation of behavior. This lack of predictability of practice for change construct to change the continuity of regular physical activity was also confirmed in the study of Hayes *et al.* [18].

However, in the study of Nahar *et al.*, the construct of practice for change was reported to change the predictor of continued physical activity among students [31]. Having a notebook can be a valuable way to organize thoughts and record the best ideas needed to progress in work and life. It seems that in a culture like Yazd, where people are not very accustomed to writing their actions in personal notebooks, actions such as writing down daily sports activities do not play an important role in the sustainability of regular physical activity. Changes in the social environment include the support of family members and health care providers who help the individual perform the behavior.

In the present study, changes in the social environment construct significantly predict the substance of regular physical activity. This means that the more support people around the participants and family members or the health team provide, the more likely the person will continue regular physical activity. This finding is consistent with the study of Hayes *et al.* [18] and Nahar *et al.* [31]. This finding is in line with the study of Khajavi *et al.*, which indicated that social support is a strong predictor of physical activity [39], and the study of Borji and Motaghi [40] which showed a significant relationship between physical activity and social support.

In the latter study, as the perceived social support of the elderly increases, so does their physical activity. Social support is a person's perception that he or she is being cared for by others. From their point of view, the person is valuable, and if he or she gets into trouble, people like friends and colleagues will help him or her. Social support refers to the sense of belonging, being accepted, loved, needed, and appreciated. Social support creates a secure relationship for each person, and the feeling of love and closeness is one of the main features of these relationships [41]. Sarason divided the dimensions of social support into five categories: emotional support, social network support, self-esteem support, instrumental support, and information support. Emotional support is the skill of getting help from others when stress increases.

The social network support reduces the stress or mental pressure by accessing the network membership. Self-esteem support means that others believe in a person with special capabilities. Instrumental support is access to financial and service resources when a person needs them to adapt to stressful events. Information support is the provision of information to understand stressful events [42]. If a woman has enough social support, for instance, her husband prepares a pool ticket and takes her to the pool, or her husband or friends accompany her on a walk, or if health care providers have training and recommendations on doing pelvic floor muscle strengthening exercises, these supports can play a very important role in perpetuating regular physical activity-related behavior. In the current study, the initiation of regular physical activity behaviors was a significant predictor of lower quality of life.

This appears to be true because until the behavior becomes institutionalized in a person, it cannot affect the control of complications and improve the quality of life. In addition, initiating regular physical activity behavior may be stressful at first because the person may not be used to doing that behavior, and the stress caused by it can reduce the quality of life. However, the sustainability of regular physical activity behavior was a significant predictor of higher quality of life, and in explaining this finding, it can be stated that when a person has regular physical activity, and this behavior becomes a habit, the conduct of doing the behavior becomes easier for them. He or she knows how to include time for regular physical activity in his or her daily plan; now the positive effect of regular physical activity on his or her quality of life is realized.

One of the strengths of this research is regarding the beneficial physical activities to improve menopausal symptoms, *i.e.*, walking, pelvic floor muscle strengthening exercise, and swimming. Furthermore, members of the research team assisted participants in completing the questionnaire, which decreased the risk of incomplete questionnaires in participants with low literacy levels. However, one of the limitations of this research is the self-reporting of the collected information, which influences the accuracy of the results. Moreover, the lack of menopausal status in the SIB system (Integrated health system in Iran) was another restriction of this research that extended the sampling process.

## CONCLUSION

The MTM is a useful model for predicting the initiation and sustainability of regular physical activity behaviors in post-menopausal women. Behavioral confidence and change in the physical environment constructs played a significant role in predicting the initiation of behavior. In addition, emotional transformation and changes in the social environment constructs are significant predictors in sustaining regular physical activity behaviors. Therefore, using this model in designing educational interventions may be very useful for this target population.

## ACKNOWLEDGMENTS

### Conflict of interest

The authors declare no conflict of interest.

### Ethical approval

The study was conducted following the Helsinki Declaration, and ethical approval was obtained from the Human Subject Institutional Review Board of Shahid Sadoughi University of Medical Sciences (IR.SSU.SPH.REC.1397.137).

### Consent to participate

Written informed consent was obtained from all participants in the study.

### Personal thanks

We want to thank the women who participated in the study. We would also like to thank the Health Care Centers at Shahid Sadoughi University of Medical Sciences for their help in developing the study.

### Authorship

NY and MAM contributed to conceptualizing. SJ and MS contributed to the methodology, NY and NB contributed to writing the original draft. RS, FH and NY contributed to editing the manuscript. NY contributed to data collection. SJ and MAM contributed to data curation. SJ and NY contributed to data analysis.
